# PD59 Inpatient Healthcare Costs And Neonatal Disorders: Insights From The Brazilian Public Health System

**DOI:** 10.1017/S0266462325101918

**Published:** 2025-12-29

**Authors:** Aline Toledo, Julia Raffin Moura, Emilie Batista Freire, Henry Maia Peixoto, Everton Nunes da Silva, Silvana Alves Pereira, Rodrigo Luiz Carregaro

## Abstract

**Introduction:**

Neonatal disorders are a significant contributor to years lost to premature mortality and years lived with disability worldwide. Healthcare cost estimates for neonatal inpatient health care and the health conditions that most affect newborns in Brazil remain unknown at the national level. We estimated the inpatient healthcare costs in Brazil and identified the most burdensome health conditions.

**Methods:**

This population-based descriptive study was conducted from the perspective of the Brazilian public health system. We used secondary nationwide data from the Hospital Information System. Inpatient healthcare records between 2011 and 2022 for newborns from zero to 27 days of life were included. Inpatient healthcare costs consisted of reimbursements for hospital procedures and daily hospital stays, including neonatal intensive care unit (NICU) admissions. Costs were adjusted for the 2023 inflation rate and presented in USD considering the parity index of 2023. The main health conditions are presented as International Classification of Diseases, 10th Revision categories.

**Results:**

A total of 3,835,128 neonatal hospital admissions accounted for approximately USD6 billion, of which 26.7 percent were for NICU admissions. The ten costliest health condition categories accounted for 75.6 percent of total inpatient healthcare costs. Although the most prevalent neonatal health condition was neonatal jaundice (n=652,314), the category of disorders related to prematurity accounted for most inpatient neonatal care costs at USD1.7 billion (USD1.1 billion for NICU), followed by neonatal respiratory distress syndrome at USD1.6 billion (USD1.2 billion for NICU), and the category of other respiratory disorders at USD316.1 million (USD238.3 million for NICU).

**Conclusions:**

This study revealed the economic burden of neonatal inpatient healthcare in Brazil, driven predominantly by NICU admissions for prematurity and respiratory conditions. The findings highlight the importance of targeted health interventions and policies to improve neonatal outcomes and optimize resource allocation.

